# RNA sequencing and bioinformatics analysis of differentially expressed genes in the peripheral serum of ankylosing spondylitis patients

**DOI:** 10.1186/s13018-023-03871-w

**Published:** 2023-05-30

**Authors:** Yongchen Bie, Xiujun Zheng, Xiaojiong Chen, Xiangyun Liu, Liqin Wang, Yuanliang Sun, Jianqiang Kou

**Affiliations:** 1 Department of Spinal Surgery, The Affiliated Hospital of Qingdao University, Qingdao, 266000 Shandong China; 2 Department of Rheumatology, The Affiliated Hospital of Qingdao University, Qingdao, 266000 Shandong China

**Keywords:** Differentially expressed genes, RNA sequencing, Serum, Ankylosing spondylitis, Biomarker

## Abstract

**Background:**

Ankylosing spondylitis (AS) is a chronic progressive autoimmune disease characterized by spinal and sacroiliac arthritis, but its pathogenesis and genetic basis are largely unclear.

**Methods:**

We randomly selected three serum samples each from an AS and a normal control (NC) group for high-throughput sequencing followed by using edgeR to find differentially expressed genes (DEGs). Gene Ontology (GO), Kyoto Encyclopedia of Genes and Genomes, Reactome pathway analyses, and Gene Set Enrichment Analysis were used to comprehensively analyze the possible functions and pathways involved with these DEGs. Protein–protein interaction (PPI) networks were constructed using the STRING database and Cytoscape. The modules and hub genes of these DEGs were identified using MCODE and CytoHubba plugins. Reverse transcription-quantitative polymerase chain reaction (RT-qPCR) was used to validate the expression levels of candidate genes in serum samples from AS patients and healthy controls.

**Results:**

We successfully identified 100 significant DEGs in serum. When we compared them with the NC group, 49 of these genes were upregulated in AS patients and 51 were downregulated. GO function and pathway enrichment analysis indicated that these DEGs were mainly enriched in several signaling pathways associated with endoplasmic reticulum stress, including protein processing in the endoplasmic reticulum, unfolded protein response, and ubiquitin-mediated proteolysis. We also constructed a PPI network and identified the highly connected top 10 hub genes. The expression levels of the candidate hub genes *PPARG*, *MDM2*, *DNA2*, *STUB1*, *UBTF*, and *SLC25A37* were then validated by RT-qPCR analysis. Finally, receiver operating characteristic curve analysis suggested that *PPARG* and *MDM2* may be the potential biomarkers of AS.

**Conclusions:**

These findings may help to further elucidate the pathogenesis of AS and provide valuable potential gene biomarkers or targets for the diagnosis and treatment of AS.

## Introduction

Ankylosing spondylitis (AS) is a chronic inflammatory arthritis and autoimmune disease with an incidence of 0.1–1.4% [1]. This disease is primarily characterized by spinal and sacroiliac arthritis [2] and is frequently associated with extra-articular features including anterior uveitis, psoriasis, or inflammatory bowel disease [3–5]. The stiffness of the affected joints becomes severe with the development of ankylosing spondylitis, leading to back pain, poor quality of life, and in more serious cases, mental illness [6, 7]. Currently, the etiology of AS is multifactorial; both environmental and genetic predispositions have been suggested to be involved in AS pathogenesis [8], including macrophage activation status, infections with particular bacteria, inflammatory cytokines, and autophagy [9–12]. Nevertheless, few genes have been shown to be associated with the disease and the actual cause of AS has remained unclear. Therefore, there is an urgent need to identify new biomarkers that can act as reliable diagnostic or prognostic indicators of AS. Such biomarkers will be invaluable in the prevention, treatment, and control of this disease.

In recent decades, ribonucleic acid (RNA) sequencing has proven to be a novel high-throughput sequencing method that uses deep-sequencing technology [13]. This approach can be used to identify abnormally spliced genes, detect allele-specific expression, and identify differentially expressed genes (DEGs). Bioinformatics analysis can use sequencing data to analyze the genome, transcriptome, and proteome information of organisms and has been used to reveal the mechanisms of disease that occurs due to abnormal biological processes at the molecular level [14, 15]. To date, multiple studies have used microarray expression and RNA sequencing to identify DEGs involved in the pathogenesis of AS [16, 17]. Peripheral blood is often considered a potential resource for the discovery of disease biomarkers [18]. However, the levels of DEGs in peripheral serum from patients with AS have not been explored, nor have these molecular mechanisms been further validated. Altered gene expression profiles that differentiate disease from healthy can be used as a basis for understanding the pathogenesis of AS.

In the current study, by expression profiling of high-throughput sequencing and experimental analysis, we identified DEGs in the serum of AS patients and normal controls. Then, a molecular mechanism of AS was proposed after analyzing these pathways and functional enrichments. Finally, with the use of these DEGs, we established the protein–protein interaction (PPI) network to identify hub genes for targeting AS.

## Materials and methods

### Patients and samples

A total of 18 patients with AS patients and 18 healthy age-matched controls were selected from the Affiliated Hospital of Qingdao University (Qingdao, China) between December 2020 and September 2021. All patients met the modified New York 1984 criteria [19] and were initially diagnosed with AS, drug-naive patients with short disease durations. None of the patients or controls had any previous history of cardiovascular disease, diabetes, hepatitis, malignancy, or other autoimmune and inflammatory illnesses.

Serum samples were collected using standard phlebotomy procedures and centrifuged at 3000 g for 10 min. The separated sera were stored in RNase-free centrifuge tubes at 80 °C until further processing. This research was approved and reviewed by the Medical Ethics Review Committee of the Affiliated Hospital of Qingdao University (approval number: QYFY WZLL 27251). All participants provided written informed consent in accordance with policies of the hospital ethics committee.

### RNA extraction and sequencing

We randomly selected three patients with AS and three normal control (NC) for high-throughput sequencing [20]. Total RNA was extracted from serum using TRIzol reagent (Invitrogen; Thermo Fisher Scientific, Inc., Waltham, MA, USA) according to the manufacturer's instructions. Subsequently, the concentration and integrity of the isolated RNA were determined using the Qubit 3.0 Fluorometer (Invitrogen, Carlsbad, CA, USA) and an Agilent 2100 Bioanalyzer (Applied Biosystems, Carlsbad, CA, USA), respectively. RNA-seq libraries were prepared using the SMARTer Stranded Total RNA-Seq kit v.2 (Takara Bio USA, Mountain View, CA, USA) as previously [21]. The RNA samples were fragmented and reversely transcribed into first-strand cDNA, followed by second-strand synthesis. After cDNA synthesis, a tailing and adapter ligation was performed, and then the cDNA was amplified by PCR. Subsequently, the cDNA library quality and concentration were evaluated using the Agilent 2100 Bioanalyzer (Applied Biosystems, Carlsbad, CA, USA). The qPCR-based KAPA Biosystems Library Quantification kit (Kapa Biosystems, Inc.) was used for the quantification of the cDNA library. Ribosomal RNA depletion was performed during library construction according to the manufacturer’s protocol. Sequencing was carried out in a 150-bp paired-end run (PE150) using the NovaSeq 6000 system (Illumina, San Diego, CA, USA).

### Analysis of DEGs

Reads were first mapped to the latest UCSC transcript set using Bowtie2 version 2.1.0 [22] and gene expression levels were estimated using RSEM v1.2.15 [23]. The trimmed mean of M-values was used to standardize gene expression. DEGs were then identified using edgeR software [24]. Genes showing altered expression with *p* < 0.05 and more than 1.5-fold changes were considered to be differentially expressed.

### Gene ontology (GO), kyoto encyclopedia of genes and genomes (KEGG), and reactome analyses

To better understand the function and pathways of DEGs in AS, we performed GO functional annotation, KEGG enrichment [25], and Reactome analyses using the R package clusterProfiler [26]. GO analysis was used to investigate biological functions based on differentially expressed coding genes. This analysis classifies functions according to the three following aspects: biological process (BP), cellular component (CC), and molecular function (MF). A lower *p*-value indicated a higher significance of a GO term (*p*-value < 0.05). The KEGG and Reactome enrichment analyses were used to predict the related pathways of each DEG. A *p*-value of < 0.05 reflected significant enrichment.

### Gene set enrichment analysis (GSEA)

GSEA was done as described in Subramanian et al. [27]. We used the R package fgsea to analyze the expression of filtered genes against MSigDB, a well-known molecular feature database. In addition, only two typical gene sets from MSigDB, H (hallmark gene sets) and C2:: CP (curated gene sets, canonical pathways), were analyzed by GSEA here. Finally, we retained results with statistical significance *p*-values < 0.05.

### Construction of a protein–protein interaction (PPI) network and identification of hub genes and key modules

To gain insights into the correlation among DEGs at the protein level, the Search Tool for the Retrieval of Interacting Genes (STRING, https://stringdb.org) database was used to construct a PPI network of these DEGs [28]. The minimum required interaction score used to construct this PPI network was 0.4, and the isolated nodes were abandoned. Cytoscape [29] was used to visualize this PPI network and we used the plug-in Cytohubba to explore the hub genes of this PPI network [30]. Based on the centrality score, the key nodes in this PPI network were determined, and then the hub genes were deduced. Simultaneously, we used the molecular complex detection (MCODE) plugin for clustering analysis of gene networks to select the key subnetwork modules.

### Validation of DEGs

Reverse transcription-quantitative polymerase chain reaction (qRT-PCR) experiments were conducted to validate the DEGs identified using high-throughput sequencing. cDNA was synthesized using the Prime Script RT reagent kit with genomic DNA eraser (TaKaRa, Tokyo, Japan), and qRT–PCR was performed on a LightCycler 480 (Roche, Indianapolis, IN, USA) using SYBR Green Master Mix (TaKaRa, Tokyo, Japan). The primer sequences are listed in Table 1. Validation experiments were performed using serum from 15 AS samples and 15 NC samples. mRNA levels of the selected new target genes were quantified by the 2^−ΔΔCt^ method after normalization to the housekeeping gene *GAPDH*.

**Table 1:** Primers used in the present study

Gene	Forward primer	Reverse primer
PPARG	GCCCTTCACTACTGTTGACTTCTCC	CAGGCTCCACTTTGATTGCACTTTG
MDM2	AGGCAGGGGAGAGTGATACAGATTC	CAGGAAGCCAATTCTCACGAAGGG
DNA2	GAAACCCAGCATCTGAAGCAAACAC	TCTCCATTTCCGAAGCAGGCATTAG
STUB1	TCCTACCTCTCCAGGCTCATTGC	ATGTCCGCCATGTACTTGTCGTG
UBTF	ATCTCCCAGAGCCAGAAGGAG	GGGAGACAGGCTCTTAACCCA
SLC25A37	CCTTCTACCGGAGCTACACCA	CCTGAGATGATGTGGGACTGC
GAPDH	GCACCGTCAAGGCTGAGAAC	TGGTGAAGACGCCAGTGGA

### Receiver operating characteristic (ROC) analyses

To assess the diagnostic value of DEGs in AS, we performed ROC analyses using the pROC R package. We calculated the area under the curve (AUC) under the binomial exact confidence interval and Ggplot2 was applied for further visualization.

### Statistical analysis

Statistical analysis was performed using SPSS 26.0 software (SPSS Inc., Chicago, IL, USA). All data are expressed as the mean ± SD. Statistical significance was determined by Student’s *t* test, and *P* values < 0.05 were considered to indicate a statistically significant difference.

## Results

### Characteristics of AS and NC patients

Table 2 presents the demographic and clinical characteristics of the 18 patients with AS and 18 NC in the present study. Patients with AS and NC were matched in terms of age and sex.

**Table 2:** Characteristics of AS patients and NC

Items	NC (*n* = 18)	AS (*n* = 18)
Age (years)	31.6 ± 7.2	32.1 ± 7.7
Gender, No. (%)		
Female	3 (16.7)	2 (11.1)
Male	15 (83.3)	16 (88.9)
CRP (mg/L)	–	15.4 ± 8.7
ESR (mm/H)	–	32.2 ± 18.9
HLA-B27 positive, No. (%)		
No	–	1 (6.6)
Yes	–	17 (94.4)
BASDAI	–	4.1 ± 1.3
BASFI	–	3.7 ± 1.2
VAS	–	5.8 ± 1.8

CRP, C-reactive protein; ESR, erythrocyte sedimentation rate; HLA-B27, human leukocyte antigen-B27; BASDAI, Bath Ankylosing Spondylitis Disease Activity Index; BASFI, Bath Ankylosing Spondylitis Functional Index; VAS, Visual Analogue Scale

### Identification of DEGs in AS

**Fig. 1:**
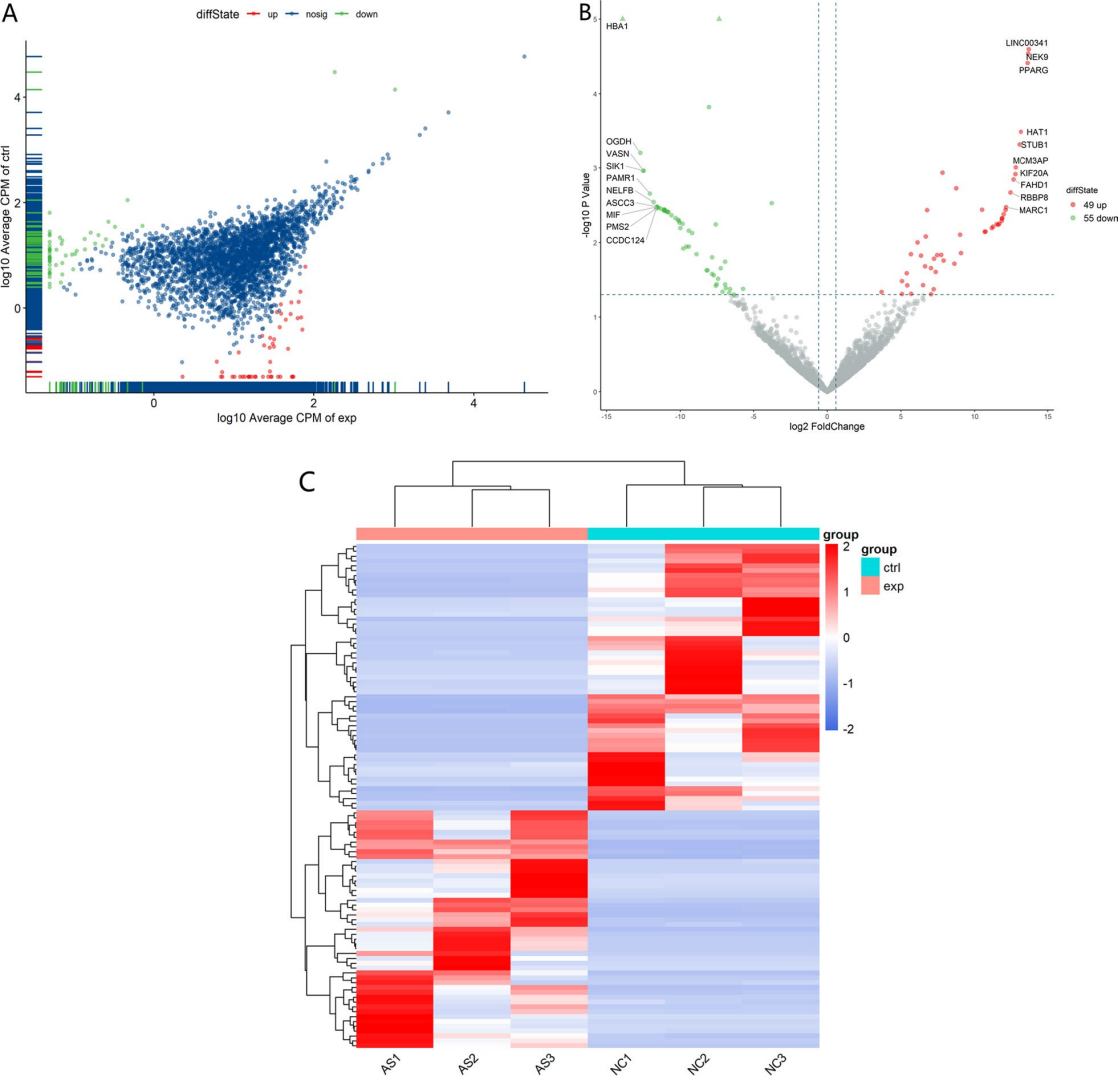
**A** scatter plot and **B** volcano plot. The red dots represent upregulated DEGs, and the green dots represent downregulated DEGs. **C** Hierarchical clustering of differentially expressed genes between the AS and control groups

In the AS Peripheral Blood Serum samples, a total of 100 DEGs were differentially expressed, including 49 upregulated DEGs and 51 downregulated DEGs, respectively. These DEGs are represented by scatter and volcano plots in Fig. 1A and B, respectively. Figure 1C shows the hierarchical clustering of these DEGs. The ten most upregulated and downregulated DEGs are shown in Table 3.

**Table 3:** Top ten upregulated and ten downregulated DEGs in AS

Gene name	logFC	Fold change	*P* value	Regulation
SYNE3	13.70024767	13,310.22803	2.53347E−05	Up
NEK9	13.66669431	13,004.23858	2.90169E−05	Up
PPARG	13.62684124	12,649.92523	3.87972E−05	Up
HAT1	13.15463256	9118.827157	0.000323846	Up
STUB	13.0556646	8514.255069	0.000478438	Up
MCM3AP	12.78714097	7068.267708	0.000980806	Up
KIF20A	12.77003424	6984.950792	0.001209986	Up
FAHD1	12.64685533	6413.318437	0.001424853	Up
RBBP8	12.43477239	5536.553204	0.002137449	Up
MARC1	12.1303543	4483.327884	0.003358303	Up
MIF	− 11.57772609	0.000327158	0.003321108	Down
ASCC3	− 11.5932764	0.00032365	0.003319781	Down
CCDC124	− 11.6024601	0.000321597	0.003347325	Down
PMS2	− 11.6352721	0.000314365	0.003485706	Down
NELFB	− 11.82545438	0.000275539	0.002867456	Down
PAMR1	− 12.07196817	0.000232261	0.002207181	Down
VASN	− 12.48849677	0.000174015	0.001089923	Down
SIK1	− 12.54756591	0.000167035	0.001085811	Down
OGDH	− 12.71646833	0.00014858	0.000627557	Down
HBA1	− 13.92287772	6.43867E-05	3.65243E−06	Down

### GO and pathway enrichment analyses

**Fig. 2:**
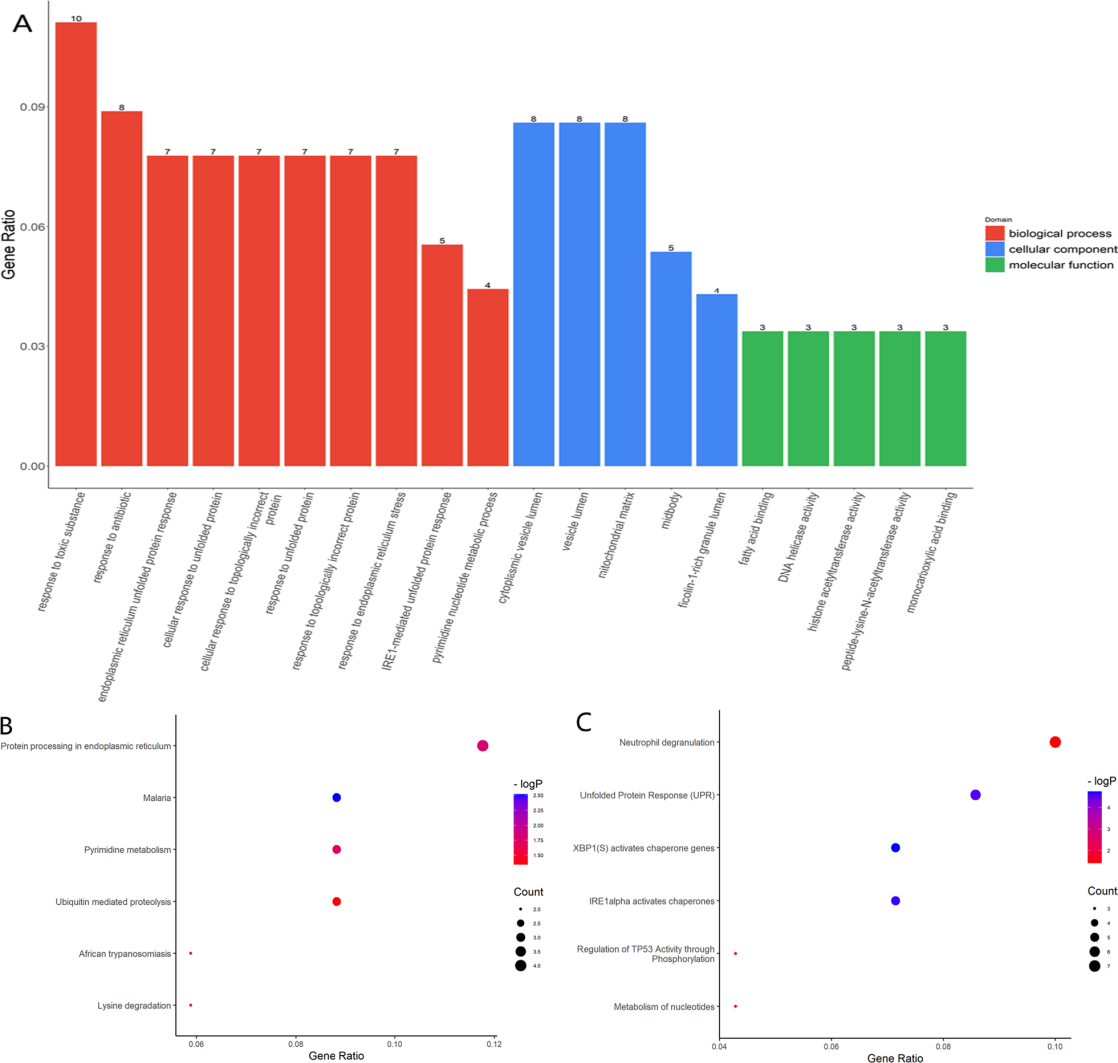

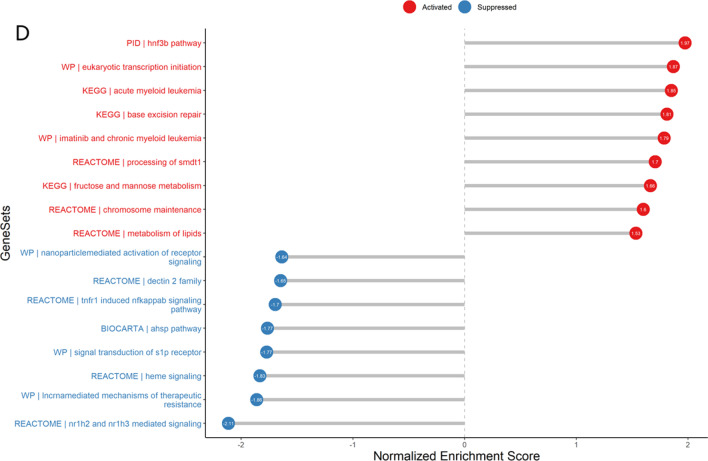
GO, KEGG, Reactome, and GSEA analyses of the differentially expressed genes in AS patient serum. **A** GO analysis. BP, CC, and MF are represented in red, blue, and green, respectively. **B** KEGG analysis. The six KEGG pathways are shown. **C** Reactome analysis. The six Reactome pathways. **D** GSEA enriched pathway. The top ten pathways (≤ 10) are shown

GO function enrichment analysis was conducted on 100 DEGs using the R package clusterProfiler, and 167 GO items with significant differences were identified, including 130 BP items, 16 CC items, and 21 MF items. It can be seen from the graph that the biological processes of BP mediated by DEGs were mainly concentrated in response to toxic substance, response to the antibiotic, and ER unfolded protein response. The results for CC were mainly concentrated in cytoplasmic vesicle lumen, vesicle lumen, and mitochondrial matrix. The results for MF showed that fatty acid binding, DNA helicase activity, and histone acetyltransferase activity were significantly enriched items (Fig. 2A).

KEGG pathway enrichment analysis of these DEGs yielded numerous signaling pathways that were significantly altered in the serum of AS. A total of six KEGG pathways were enriched for these DEGs (Fig. 2B), and protein processing in the endoplasmic reticulum (ER) was the most significantly enriched KEGG pathway associated with AS. For validating the biofunctions related to such DEGs, Reactome is another well-known signaling pathway database. We also identified six pathways based on Reactome analysis in the present study (Fig. 2C). The most enriched Reactome terms of AS were neutrophil degranulation and unfolded protein response (UPR).

### GSEA-enriched pathways

Simultaneously, we utilized GSEA to identify the potential mechanism underlying AS. We analyzed 100 top genes and identified 17 pathways that were enriched significantly. Among the pathways with the highest enrichment scores were the nr1h2 and nr1h3 mediated signaling pathways and the hnf3b pathway. We visualized the top ten pathways (≤ 10) with the most significant activation and inhibition by GSEA (Fig. 2D).

### PPI network analysis

**Fig. 3:**
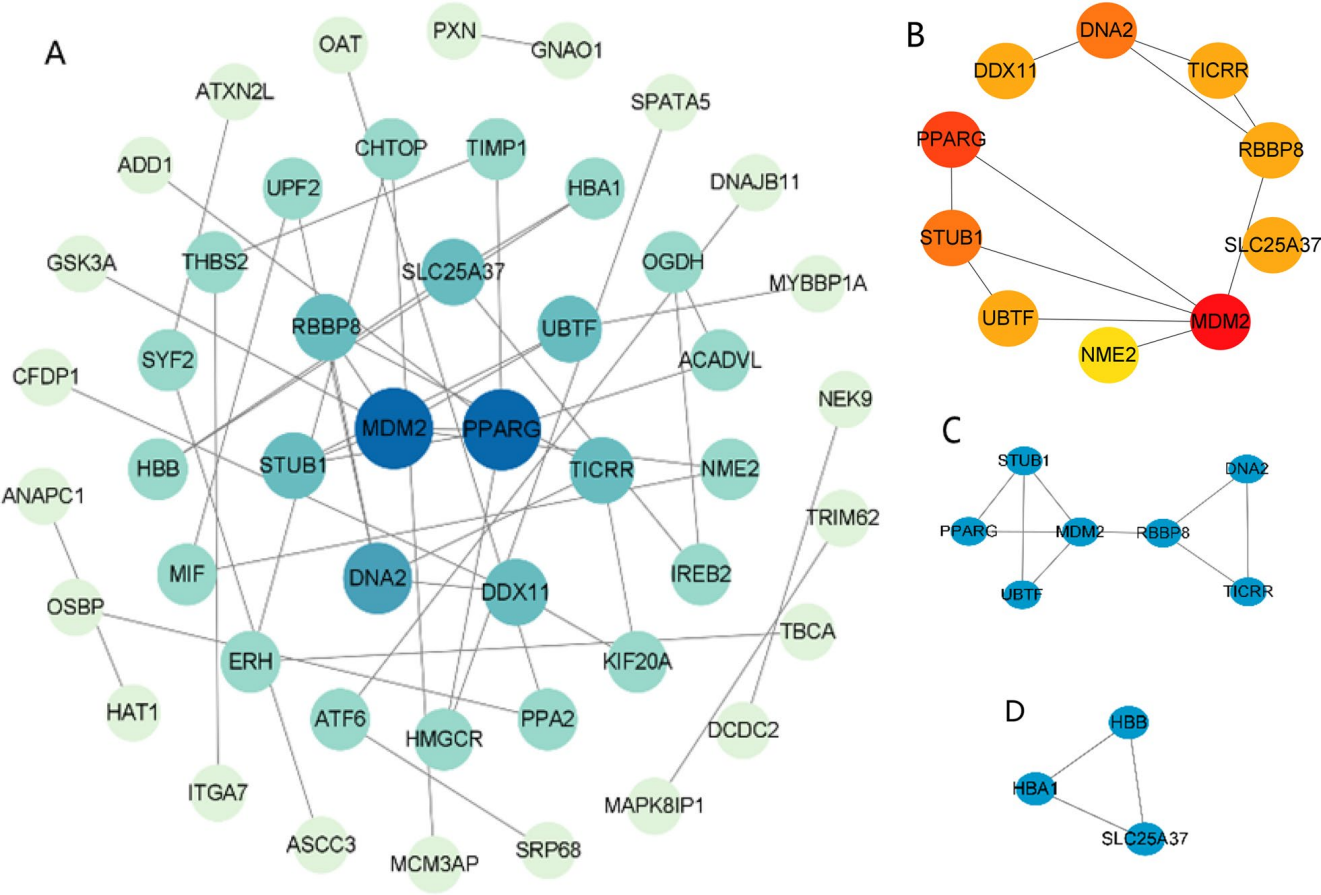
Protein–protein interaction (PPI) networks and modules. **A** PPI network of DEGs was analyzed using Cytoscape software. The size and color of the nodes corresponding to each gene were determined according to the degree of interaction. The size of the nodes reflects the degree value, where the larger the node, the greater the degree value. The closer to the blue node, the higher connectivity between two nodes. **B** PPI network for the top ten hub genes. **C** and **D** Graphic representation of top two significant modules of the PPI network. (**C** Module 1, **D** Module 2)

A total of 55 proteins and 58 edges were obtained with a cutoff value of credibility > 0.4, as shown in Fig. 3A. Isolated nodes from the PPI network were abandoned. The hub genes were selected from the PPI network of AS-related genes using the cytoHubba plugin. The results demonstrated that ten hub genes could be identified using the Maximal Clique Centrality (MCC) algorithm, including *PPARG*, *MDM2*, *DNA2*, *STUB1*, *UBTF*, *SLC25A37*, *TICRR*, *RBBP8*, *DDX11*, and *NME2*, as shown in Fig. 3B. A summary of the degree values for the ten genes is provided in Table 4. Of these, *PPARG* (degree = 6) and *MDM2* (degree = 6) were identified as the most significant genes. The key subnetwork modules were identified using the Cytoscape plug-in MCODE. According to the degree of importance, two significant modules were selected from this PPI network. The results revealed two modules as shown in Fig. 3C and D. Module 1 was composed of seven nodes and nine edges (score: 3.000), followed by module 2, which was composed of three nodes and three edges (score: 3.000).

**Table 4:** The degree values of top ten hub genes

Gene	Degree
PPARG	6
MDM2	6
DNA2	4
STUB1	3
UBTF	3
SLC25A37	3
TICRR	3
RBBP8	3
DDX11	3
NME2	2

### Verification of DEGs by qRT-PCR

**Fig. 4:**
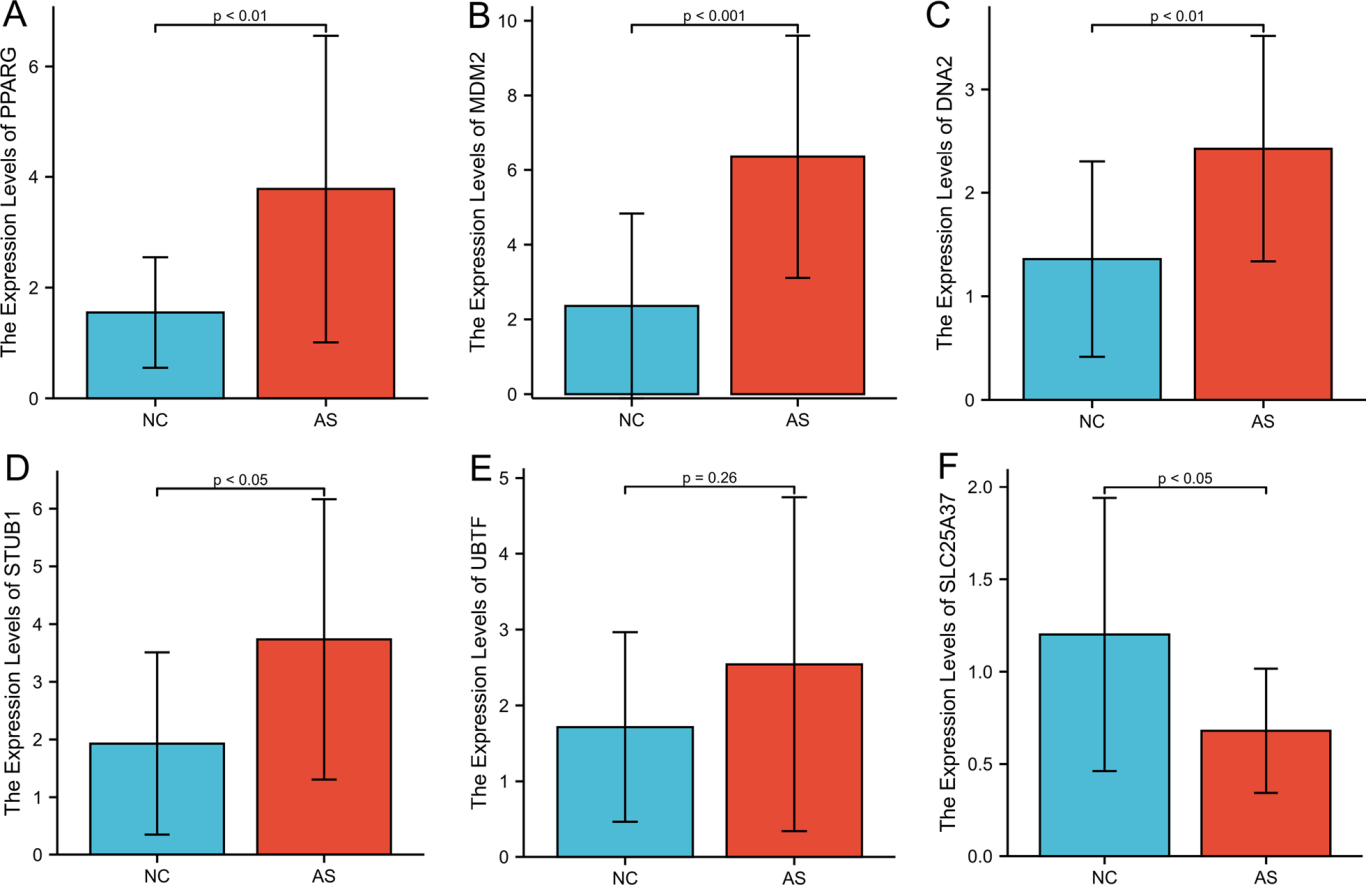
qRT-PCR-based validation of the expression of six differentially expressed genes in control and AS patient serum

To validate these major results, we selectively performed qRT-PCR analysis of six hub genes including *PPARG*, *MDM2*, *DNA2*, *STUB1*, *UBTF*, and *SLC25A37* using RNA from 15 AS patients and 15 healthy controls. The relative gene expression levels of *PPARG*, *MDM2*, *DNA2,* and *STUB1* were significantly higher in blood samples from AS patients when compared with those from healthy controls. Moreover, the expression levels of *SLC25A37* were significantly lower in the AS group relative to the healthy control group (Fig. 4).

### Receiver operating characteristics curve (ROC) analysis of confirmed DEGs in serum

**Fig. 5:**
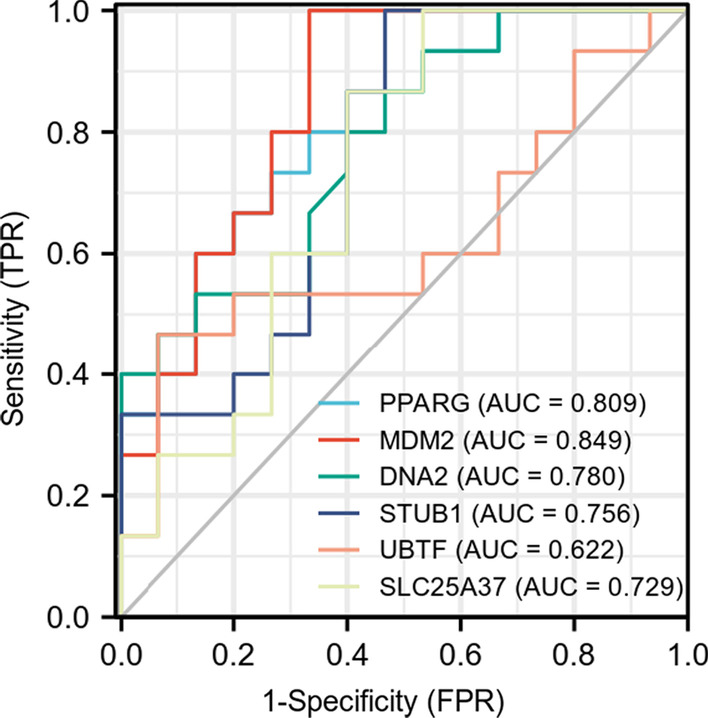
Visualization and details of the ROC curve

To explore the possibility of these six genes as diagnostic biomarkers of AS patients, our qRT-PCR data were subjected to ROC analysis to evaluate their diagnostic ability. As shown in Fig. 5, except *UBTF*, the areas under the ROCs of the other five genes were all > 0.7. This finding indicates that these genes may be sensitive biomarkers that can distinguish AS patients from individuals without AS.

## Discussion

Recently, along with the continuous development of sequencing technologies and bioinformatics technology, studies on the molecular pathogenesis of diseases using bioinformatics tools have emerged in the field of biomedicine. Genome-wide genetic screens have identified a number of genes contributing to AS, but none has yet fully explained the disease process [31]. Previous studies have found that DEGs were abundantly expressed in synovial tissue, whole blood and peripheral blood mononuclear cells in AS [32–34]. To date, there has been no research highlighting the expression profiles of DEGs in serum. Taking this into account, we sought to reveal the role of serum DEGs in the pathogenesis of AS. In the present study, we adopted high-throughput sequencing to analyze DEG profiles in serum isolated from AS patients and controls. We successfully identified 100 DEGs and then performed a range of analyses (GO, KEGG pathway, GSEA, and PPI) in an attempt to uncover novel insights into DEG functions in AS.

In this work, through annotation and functional enrichment analysis, we revealed that numerous genes associated with unfolded protein response and immunoreaction and apoptosis may play a key role in AS, GO and pathway analysis of DEGs demonstrated that the DEGs were mainly enriched in protein processing in ER, response to endoplasmic reticulum stress (ERS), UPR, neutrophil activation involved in immune response and the intrinsic apoptotic signaling pathway. Studies have confirmed that misfolding of the human leucocyte antigen B27 allele (HLA-B27) forms non-native heavy chain dimeric structures [35]. Dimers may accumulate in the ER, resulting in increased ER stress and potentially leading to the onset of pro-inflammatory responses [36]. In addition, misfolding proteins within the ER can induce the UPR, which is a cellular stress response that initiates transcriptional changes whose function is to restore ER homeostasis [37]. Very recently it was shown that M1 macrophages produce a UPR and stimulated ER stress-related IL-23 in AS patients [38]. The UPR is also associated with aberrant distribution and function of plasmacytoid dendritic cells [39]. Taken together, the results of this study in association with other previous studies have indicated a basis for the vital role of ERS and UPR in the pathogenesis and progression of AS.

Thereafter, the STRING database was used to build a PPI network and Cytoscape was used to identify significant module and hub genes [40]. Analysis of the PPI network constructed based on AS-related DEGs identified direct or indirect crosstalk between these genes. According to the MCC algorithm from the CytoHubba plugin in Cytoscape, the top ten AS-related genes were identified, including *PPARG*, *MDM2*, *DNA2*, *STUB1*, *UBTF*, *SLC25A37*, TICRR, *RBBP8*, *DDX11*, and *NME2*. A high degree value indicated that these genes play a pivotal role and modulate the functions of this network. Furthermore, a total of three cluster modules from this PPI network were extracted by MCODE analysis. Modularization contributed to analyzing the biological functions of the intricate networks of our RNA-seq data in AS. Finally, to validate the results of our bioinformatics analyses, we used qRT-PCR to determine the expression of hub genes that were related to AS, including *PPARG*, *MDM2*, *DNA2*, *STUB1*, *UBTF*, and *SLC25A37*. The results of our qRT-PCR assays provided further verification that our high-throughput sequencing results were reliable. Next, to assess the diagnostic utility of these hub genes, a ROC curve analysis was performed. The AUCs for five hub genes were more than 0.7, suggesting that these genes could effectively distinguish between samples from AS patients and normal controls. Thus, they should be novel and efficient serum indicators of AS in patients.

Several studies on these hub genes are related to the occurrence and development of autoimmune diseases. PPARG encodes a member of the peroxisome proliferator-activated receptor (PPAR) subfamily of nuclear receptors. PPARs, especially PPARG, contribute to the inhibition of key pro-inflammatory genes such as NF-kB, TNFα, TGFβ, and the interleukins IL-1a and IL-6 [41, 42]. In a very recent study of CD14+ monocytes from systemic lupus erythematosus patients, it was reported that there was an emergence of an immunosuppressive M2-phenotype upon TLR-induced epigenetic activation of PPARG expression [43]. Moreover, lipocalin 2 modulated by PPARG could be a potential pathway involved in concurrent inflammation and ankylosis in inflammatory bowel diseases and ankylosing spondylitis [44]. MDM2 is a multi-functional protein that is involved in both the p53 and NF-κB signaling pathways [45]. DNA induction of MDM2 promotes proliferation of human renal mesangial cells and alters peripheral B cell subsets in pediatric systemic lupus erythematosus [46]. Another study found a potential association between the del1518 variants in MDM2 and rheumatoid arthritis and indicates that combinatorial genotypes and haplotypes in the *MDM2* locus may be linked to rheumatoid arthritis [47]. These hub genes thus regulate immune-related diseases and have the potential to serve as diagnostic and therapeutic targets in these diseases.

Although potential DEGs in AS were identified based on bioinformatics, there were still some limitations to this study. First, the number of samples in this study was limited. Second, further in vitro and in vivo experiments to validate the DEGs and their potential mechanisms are lacking. Therefore, further research is warranted to address the possible limitations of this study in terms of biased results and conclusions.

## Conclusion

Using high-throughput RNA sequencing, we analyzed the DEGs profile in NC and AS groups in serum tissue. Many of these DEGs were enriched in several signaling pathways associated with ERS, which can provide novel clues for understanding the mechanism driving AS. In addition, we found that PPARG and MDM2 can be used as novel potential molecular targets for the diagnosis and treatment of AS. In conclusion, our results may provide a theoretical basis for further studies to elucidate the molecular mechanism of AS and provide more therapeutic targets for future clinical interventions.

## Data Availability

The datasets generated and/or analyzed during the current study are available in the Gene Expression Omnibus (GEO) repository, under accession number GSE205812 are publicly accessible at http://www.ncbi.nlm.nih.gov/geo.
